# Activation of Nell-1 in BMSC Sheet Promotes Implant Osseointegration Through Regulating Runx2/Osterix Axis

**DOI:** 10.3389/fcell.2020.00868

**Published:** 2020-09-22

**Authors:** Kaichen Lai, Yue Xi, Xue Du, Zhiwei Jiang, Yongzheng Li, Tingben Huang, Xiaoyan Miao, Huiming Wang, Ying Wang, Guoli Yang

**Affiliations:** ^1^The Affiliated Stomatology Hospital, Zhejiang University School of Medicine, Hangzhou, China; ^2^Key Laboratory of Oral Biomedical Research of Zhejiang Province, Zhejiang University School of Stomatology, Hangzhou, China

**Keywords:** implant osseointegration, cell sheet and tissue engineering, OPG-RANKL axis, Runx2, Osterix, NELL-1

## Abstract

Neural epidermal growth factor-like 1 protein (Nell-1) is first studied because of its association with human craniosynostosis. Nell-1 has been used to accelerate the process of fracture healing because of the osteoinductive ability in recent years. However, the role of Nell-1 during the process of osteointegration is unknown. Here we show that activation of Nell-1 in the BMSC sheet promotes osseointegration *in vivo* and *in vitro*. We found that overexpression of Nell-1 improved osteogenic differentiation and enhanced matrix mineralization of BMSCs through increasing expression of Runx2 and Osterix. Activation of Nell-1 up-regulated the expression ratio of OPG/RANKL, which might have a negative influence on osteoclast differentiation. Furthermore, we obtained BMSC sheet-implant complexes transfected with lentivirus overexpressing and interfering Nell-1 in *in vivo* study, and confirmed that overexpression of Nell-1 promoted new bone formation around the implant and increased the bone-implant contacting area percentage. Our results demonstrate that activation of Nell-1 improves implant osteointegration by regulating Runx2/Osterix axis and shows the potential of BMSC sheet-implant complexes in gene therapy.

## Introduction

In recent years, the demands for oral implantation increase gradually. In order to shorten treatment time and broaden indications, plenty of studies focus on implant surface modification in order to promote bone marrow mesenchymal stem cells (BMSCs) osteogenic differentiation and accelerate the formation of implant osseointegration (Lai et al., 2017; Jiang et al., 2018; Ko et al., 2020). Among them, the sandblasted, large-grit, acid-etched (SLA) Ti implants are particularly representative (Dong et al., 2017). Moreover, a lot of research focuses on implant surface with biological activities, which assembles proteins or peptides on implant surfaces (Chen et al., 2018). However, proteins and peptides are inactivated easily and may induce immune response so that they are difficult to be popularized clinically. On the contrary, gene therapy is targeted without triggering an immune response. Hence we plan to find a proper gene and assemble it on implant surface to promote implant osseointegration.

Neural epidermal growth factor-like 1 protein (Nell-1) is first studied because of its association with human craniosynostosis (CS) (Ting et al., 1999). It has been verified that the transgenic mice overexpressing Nell-1 shows a CS-like phenotype (Zhang et al., 2002), and the Nell-1 deficient mice shows bone defects in cranium and vertebra (Jayashree et al., 2006). Studies have compared the osteoinductive potential of Nell-1 with BMP-2 and find that the bone tissue where Nell-1 inducing is denser, more calcified, and the positioning is more accurate (Yuan et al., 2013). Further research has demonstrated that Nell-1 partially recuses the bone defect due to knocking out Runx2 partially. What is more, studies have reported that Nell-1 regulates the phosphorylation of Runt-related transcription factor-2 (Runx2) (Truong et al., 2007), and is a direct transcriptional target of Osterix (Chen et al., 2011). It has been verified that Osterix is a downstream gene of Runx2 and there are binding sites between them. Therefore, we take Runx2 and Osterix as an axis in this study. However, it is unclear whether Nell-1 regulates Runx2/Osterix axis during the process of implant osseointegration.

Recently, researchers have proposed that Nell-1 not only enhances bone formation, but also antagonizes inflammatory reactions which may inhibit osteoclast differentiation (Shen et al., 2013; Li et al., 2020). Osseointegration, which contains biological activities of osteoblast and osteoclast, is a complicated dynamic process. Several reports have showed that osteoprotegerin (OPG) and receptor activator of nuclear factor kappa B ligand (RANKL) (Pang et al., 2015) play important roles in modulating the cross-talk between osteoblasts and osteoclasts.

The aim of this study is to prepare Nell-1 modified BMSC sheets on the surface of implants to explore the effect of Nell-1 on promoting implant osseointegration, and to characterize the possible mechanism.

## Materials and Methods

### Isolation and Culture of Rat BMSCs

This study was approved by the Institutional Animal Care and Use Committee of Zhejiang University, Hangzhou, China. In this study, Male Sprague-Dawley rats (age: 4 weeks old) were employed. The isolation and culture of the BMSCs were performed as previously described (Donald and Arnold, 2006). BMSCs were cultured in basal medium including α-MEM (BI, Israel) containing 10% fetal bovine serum (FBS; Gibco, United States), 0.272 g/L L-glutamine (Sigma, United States), 1% penicillin (Gibco, United States), and 1% streptomycin (Gibco, United States).

### Osteogenic Potential of BMSCs Transfected With Nell-1

Lentiviral vector overexpressing and interfering Nell-1 (Rat, NM_81733) and the lentiviral vector negative control (LVNC) were prepared by GenePharma (Shanghai, China). BMSCs were cultured in 24-well plates transfected with corresponding lentiviral. At 7th and 14th day, samples were collected, and a phosphatase substrate kit (Wako, Japan) was used to quantify ALP activity. Osteocalcin (OCN) was a biochemical marker of bone formation. An osteocalcin ELISA kit (R&D System Inc., United States) was applied to detect the release of OCN at 7th, 14th, and 21th day. BMSCs transfected with corresponding lentiviral were cultured in 6-well plates for 21 days and stained by using Alizarin Red (AR) assay to evaluate the matrix mineralization qualitatively. Real-time reverse transcription-polymerase chain reaction (RT-PCR) analysis and western blot analysis were executed at 7th, 14th, and 21th day to assess the expression of ALP, OCN, Nell-1, Runx2, Osterix at gene and protein levels. The sequences of PCR primers for ALP, OC, Nell-1, Runx2, Osterix (Sangon Biotech, Shanghai, China) were presented in Table 1. The primary antibodies for rat Nell-1, Runx2, Osterix (Abcam, United Kingdom) and β-actin (Wuhan Goodbio Technology Co., Ltd.) (1:1000 diluted) were used in this study.

**TABLE 1:** Nucleotide sequence for RT-PCR primers.

Gene	Sequence of primer (5′-3′)
GAPDH, rat	Forward: ACAGCAACAGGGTGGTGGAC
	Reverse: TTTGAGGGTGCAGCGAACTT
ALP, rat	Forward: TGGTACTCGGACAATGAGATGC
	Reverse: GCTCTTCCAAATGCTGATGAGGT
OCN, rat	Forward: AGGGCAGTAAGGTGGTGAATAGA
	Reverse: GAAGCCAATGTGGTCCGCTA
Nell-1, rat	Forward: CGGGTTGTATCGCTGTGAC
	Reverse: CAGAATGCTTTGCAGATGGTG
Runx2, rat	Forward: CAGTATGAGAGTAGGTGTCCCGC
	Reverse: AAGAGGGGTAAGACTGGTCATAGG
Osterix, rat	Forward: CTGGGAAAAGGAGGCACAAAGA
	Reverse: GGGGAAAGGGTGGGTAGTCATT
OPG, rat	Forward: ATGAACAAGTGGCTGTGCTG
	Reverse: TAAGAGTGGTCAGGGCAAGG
RANKL, rat	Forward: GTACTTTCGAGCGCAGATGG
	Reverse: TCCAACCATGAGCCTTCCAT

### Nell-1 Regulating Runx2/Osterix Axis

Lentiviral vector interfering Runx2 and Osterix were prepared by GenePharma (Shanghai, China). BMSCs were transfected with lentiviral in different order (overexpressing Nell-1 first and then interfering Runx2 or Osterix, interfering Runx2 or Osterix first and then overexpressing Nell-1) and cultured at 6-well plates. PCR analysis and western blot analysis were taken at 14th day to evaluate the regulation of Nell-1 on expression of Runx2/Osterix axis.

### The Regulation of Nell-1 Transfected BMSCs on OPG and RANKL Expression

Osteoprotegerin and RANKL are cytokines secreted by BMSCs and involved in the regulation of osteoclast differentiation. BMSCs were transfected with lentiviral overexpressing Nell-1 and cultured at 6-well plates. PCR analysis and western blot analysis were taken at 7th day to evaluate the effect of Nell-1 on osteoclast differentiation. The sequences of PCR primers for OPG, RANKL (Sangon Biotech, Shanghai, China) are presented in Table 1.

### Preparation and Vitality of BMSC Sheets

Light-controlled method was used to obtain BMSCs sheets. A TiO2 nanodot (TN) was prepared on a quartz substrate (1 cm × 1 cm) as previously reported by Hong et al. (2013). In a 24-well plate, 3 × 10^4^ BMSCs transfected with lentiviral were seeded per TiO2 nanodot film with 1.2 μg/mL fibronectin (Sigma, United States). After cultured for 5 days, BMSC sheets were formed and desorbed from quartz wafers by illumination under 5.5 mW/cm^2^ ultraviolet (UV) light at 365 nm for 30 min.

To detect whether UV light had a negative effect on cell viability, BMSC sheets were reseeded on a 24-well plate. A live-dead staining kit was used to measure the viability of BMSC sheets. Thirty percent ethanol treated BMSC sheet was used as a control group. In addition, the release of 8-hydroxy-2′-deoxyguanosine (8-OH-dG) was measured by using an ELISA kit (Cusabio, CSB-E10526r) to estimate DNA damage of BMSC sheets. Cytotoxicity test was executed using the cell counting kit-8 (CCK8, Dojindo, Japan) at specified time points. BMSC sheets digested by trypsin was used as a control group. Re-adhesion ability was also measured. An inversed optical microscope was used to observe cell sheets adhesion and BMSCs migration.

### Preparation and Implantation of BMSC Sheet-Implant Complex

**FIGURE 1:**
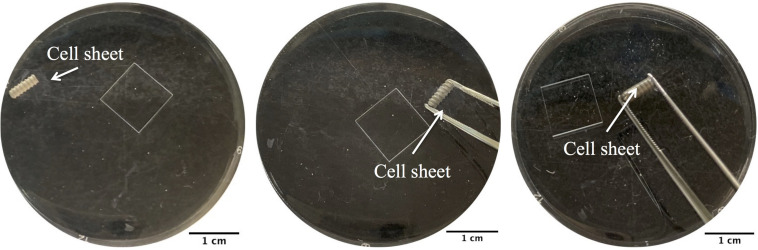
Experimental procedures for the assembly of BMSC sheet-implant complexes.

The implants used in this study were screw type with length of 6.0 mm and outside diameter of 2.2 mm. The implants were grit-blasted and acid etched as previously described (Yang et al., 2009). Types of cell sheets (Nell+ BMSC sheets, Nell− BMSC sheets, LVNC BMSC sheets, BMSCs sheets) were prepared using the transfected BMSCs. The different types of BMSC sheets were floated in 1x PBS, and then SLA implants under the floating cell sheets were picked up (Figure 1). The BMSC sheet-implant complexes were cultured in an incubator for 30 min with a blob of fresh medium. Then, they were cultured in basal medium for 1 week.

All Institutional and National Guidelines for the care and use of animals were followed. The *in vivo* study was approved by the Institutional Animal Care and Use Committee of Zhejiang University (Grant No.: ZJU20160317), Hangzhou, China. Ninety male Sprague-Dawley rats, aged 3 months, were used for osseointegration measurement at specified time points after surgery (*n* = 6/group/time points, SLA group, Blank BMSC sheets group, Nell+ BMSC sheets group, Nell− BMSC sheets group, LVNC BMSC sheets group). Implants were placed into the medial aspect of the distal tibia close to metaphysis. In order to compare the osseointegration ability of different groups, 30 rats were sacrificed at 4th, 8th, and 12th week.

### Analysis of Osseointegration

The tibias with implants were obtained after 4 weeks, 8 weeks, and 12 weeks, and fixed in 4% PFA for 24 h. A Micro-CT (μCT-100; Scanco Medical AG, Switzerland) was used to scanned the samples. After micro-CT analysis, the samples were cut into slices along the long axis of implants. The slices were stained with Gieson’s picro-fuchsin and Stevenel’s blue. The percentage of bone volume/total tissue volume on the inner of the threads (BV/TV) and the bone-implant contacting area percentage (BIC) in the threads along the surface of implant were measured.

### Statistical Analysis

Each experiment of BMSCs repeated at least three times. All of statistical analyses were performed with SPSS (version 22.0; Chicago, IL, United States). A one-way ANOVA test followed by a SNK *post hoc* test was executed to confirm whether statistical differences between each group exist. *P* < 0.05 was taken for statistical difference.

## Results

### Osteogenic Ability of Nell-1 Transfected BMSCs

**FIGURE 2:**
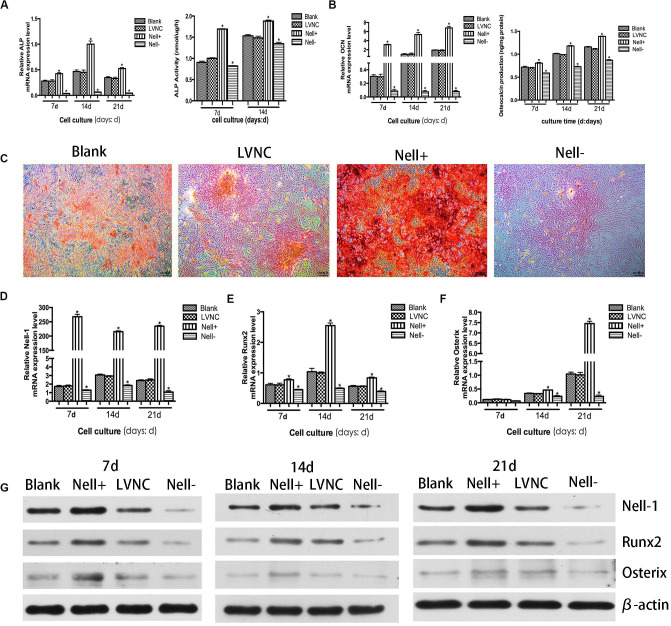
Osteogenic potential of Nell-1 gene. BMSCs transfected with lentiviral vector overexpressing Nell-1 (Nell+) demonstrated higher ALP activity **(A)** and more osteocalcin production **(B)**. **(C)** Alizarin Red staining after 21 days of osteogenic induction. *P < 0.05 compared with Blank group. Overexpression of Nell-1 improve the expression levels of Nell-1 **(D)**, Runx2 **(E)**, and Osterix **(F)** at gene and protein **(G)** levels. Interfere of Nell-1 reveled a converse result. *P < 0.05 compared with blank group.

In order to observe the effect of Nell-1 on osteogenic ability of BMSCs, ALP activity analysis, OC release analysis, extracellular matrix (ECM) mineralized nodule staining, RT-PCR analysis, and western blot analysis were executed. Figures 2A,B showed that the activity of ALP and the OC protein release significantly increased in the Nell+ group, while in the Nell− group, they decreased significantly. Moreover, compared with the blank group and the LNVC group, the mRNA levels of ALP and OC were up-regulated in the Nell+ group and down-regulated in the Nell− groups in accord with the expression in protein level.

The matrix mineralization occurred at the late stage of bone formation. Compared with the blank group and the LVNC group, more positively stained cell colonies and denser staining were found in the Nell+ group. Meanwhile, cells of Nell− group presented lighter staining (Figure 2C). Furthermore, to verify the effects of Nell-1 (Figure 2D) on osteoblastic differentiation of BMSCs, the expression of Runx2 and Osterix was evaluated. The results of RT-PCR (Figures 2E,F) and western blot (Figure 2G) revealed that the expression of Runx2 and Osterix significantly increased in the Nell+ group and decreased in the Nell− group at gene and protein levels.

### Analysis of Nell-1 Regulating Runx2/Osterix Axis

**FIGURE 3:**
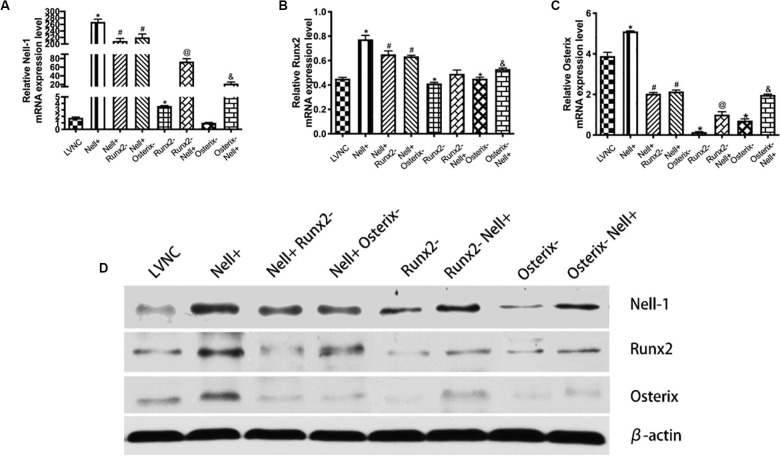
Identification of underlying regulation mechanism between Nell-1 and Runx2/Osterix axis in 7th day. **(A–C)** BMSCs are transfected with lentiviral vector (LVNC), lentiviral vector overexpressing Nell-1 (Nell+), lentiviral vector overexpressing Nell-1 first and then lentiviral vector interfere Runx2 (Nell+ Runx2–), lentiviral vector overexpressing Nell-1 first and then lentiviral vector interfere Osterix (Nell+ Osterix–), lentiviral vector interfere Runx2 (Runx2–), lentiviral vector interfere Runx2 first and then lentiviral vector overexpressing Nell-1 (Runx2– Nell+), lentiviral vector interfere Osterix (Osterix–), and lentiviral vector interfere Osterix first and then lentiviral vector overexpressing Nell-1 (Osterix– Nell+). The **(A–C)** RT-PCR and **(D)** western blot analysis of Nell-1, Runx2, and Osterix. *P < 0.05 compared with LVNC group. ^#^*P* < 0.05 compared with Nell+ group. ^@^*P* < 0.05 compared with Runx2– group. ^&^*P* < 0.05 compared with Osterix– group.

To analyze the relationship between Nell-1 and Runx2/Osterix axis further, the Nell-1 gene was overexpressed and the Runx2 gene and the Osterix gene were interfered (Figure 3A). The results in Figures 3B,C showed that the expression level of Runx2 and Osterix increased after overexpressing Nell-1 at 7th day. Inhibition of Runx2 and Osterix after overexpression of Nell-1 reduced the expression of Nell-1, Runx2, and Osterix. When the expression of Runx2 was inhibited, expression of Nell-1 and Osterix were down-regulated. Overexpression of Nell-1 rescued the expression of Nell-1 and Osterix, but the expression of Runx2 had no significant difference. The expression of Nell-1 and Runx2 decreased after inhibition of Osterix. The expression of Nell-1, Runx2 and Osterix were rescued after overexpressing Nell-1. The protein expression of Nell-1, Runx2, and Osterix was in accord with the expression of mRNA level (Figure 3D).

### Analysis of Nell-1 Regulating Expression Ratio of OPG/RANKL

**FIGURE 4:**
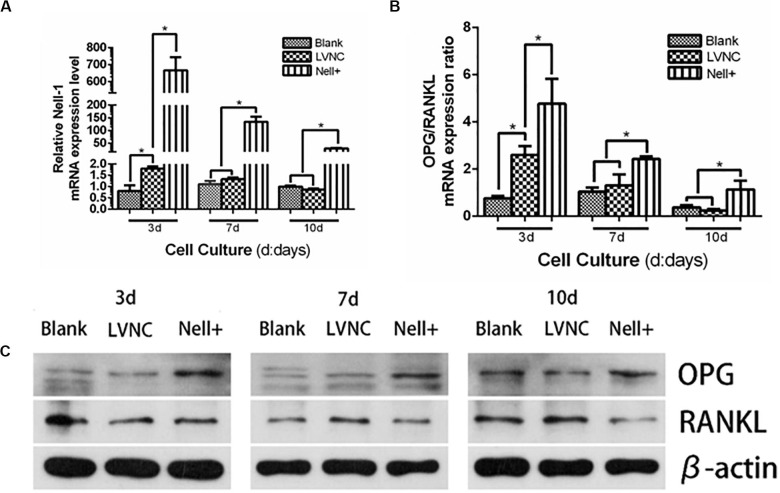
Analysis of Nell-1 regulating osteoclast differentiation. **(A–C)** Overexpression of Nell-1 improve the mRNA and protein expression ratio of OPG/RANKL. *P < 0.05.

To explore the effect of Nell-1 regulating the osteoclast differentiation, the expression of OPG/RANKL ratio was measured. The results in Figure 4 showed that overexpressing Nell-1 raised the gene expression ratio of OPG/RANKL, meanwhile the protein expression of OPG was increase and the protein expression of RANKL was decline.

### Viability of BMSC Sheet

**FIGURE 5:**
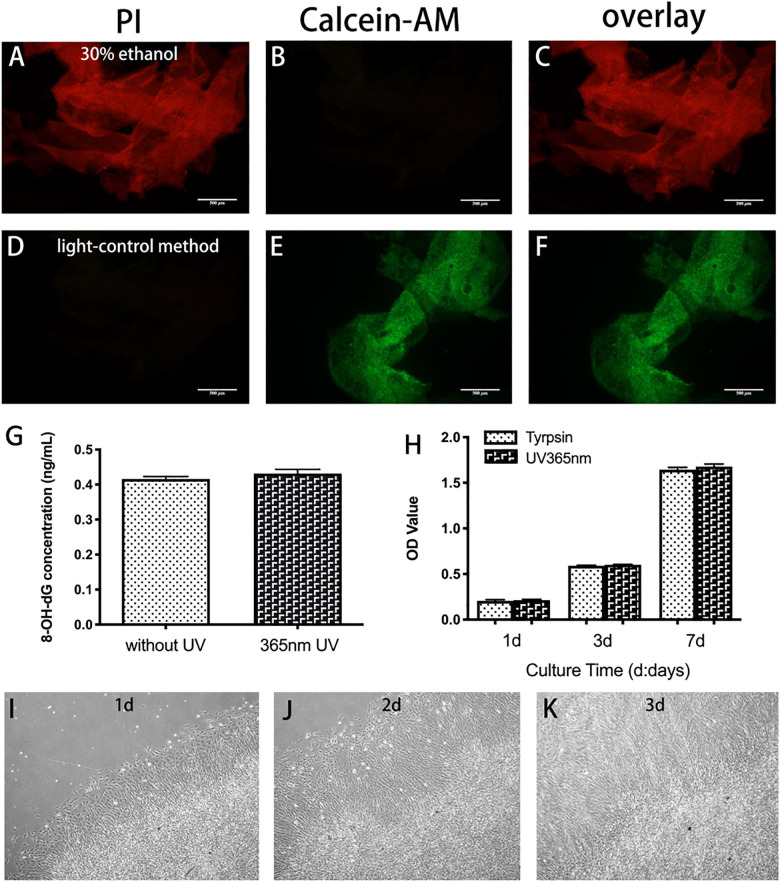
Viability of BMSC sheet generated by light-control method. **(A–F)** Live-dead staining of BMSC sheet. Calcein-AM is used to stain with live cells (green, **B,E**) and PI is used to stain with dead cells (red, **A,D**). Compared with 30% ethanol treated group, fewer dead cells are found in the light-control group. **(G)** Release of 8-OH-dG in the 365 nm UV group and no UV group. **(H)** Evaluation of the viability of BMSCs sheets generated by trypsin and light-control group using CCK8 assay. **(I–K)** Re-attachment to plate of BMSC sheets generated by light-control in day 1 **(I)**, day 2 **(J)**, and day 3 **(K)**. Scale bar: 500 μm.

The results of live-dead staining demonstrated that BMSC sheets had good viability (Figures 5D–F), while BMSC sheets treated with 30% ethanol demonstrated almost all apoptosis (Figures 5A–C). The concentration of 8-OH-dG was detected to determine whether UV light had a negative effect on BMSCs sheets. Figure 5G showed that UV light did not cause DNA damage of cells. The results of CCK-8 assay demonstrated that BMSCs sheets generated by light-control group maintained strong proliferative ability (Figure 5H), which was consistent with live-dead staining. Furthermore, BMSC sheets obtained by UV light illumination re-adhered to 24-well in the 1st day (Figure 5I), and cells removed from the sheets had overspread 24-well in the 3rd day (Figure 5J,K).

### Evaluation of Osseointegration

**FIGURE 6:**
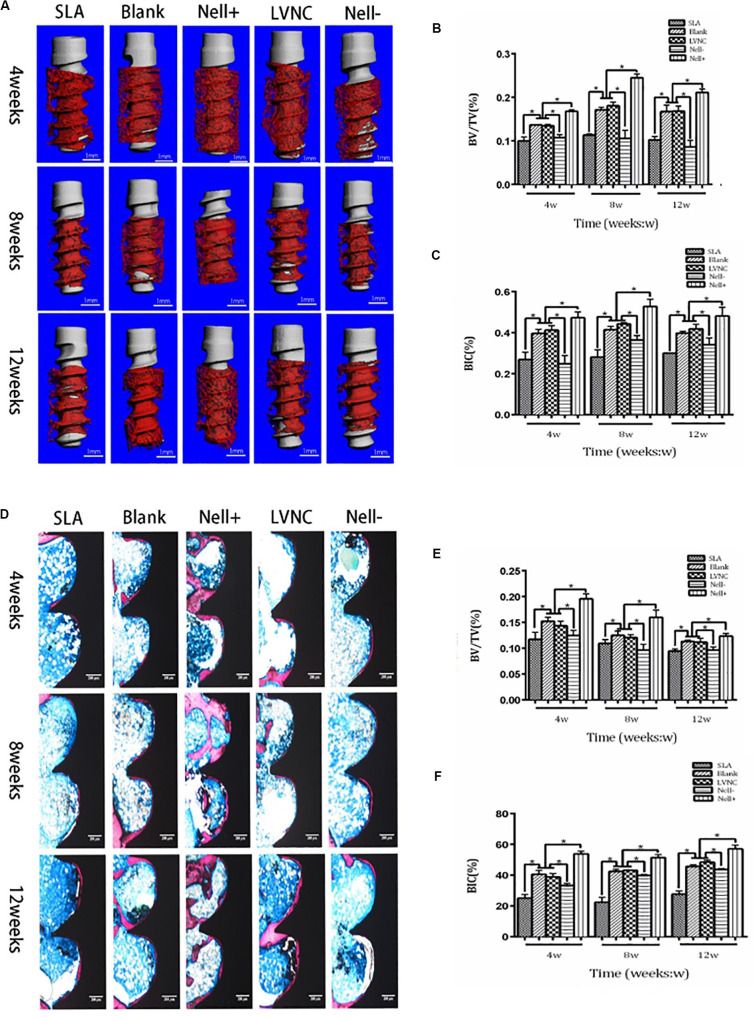
Five groups of implants (SLA group, Blank group, Nell+ group, LVNC group, and Nell– group) are inserted into the tibiae of rats and analyzed by Micro-CT and hard tissue sections. **(A)** after 4, 8, and 12 weeks of healing. **(B)** Total bone volume around the implants, expressed as the index of the bone volume per total volume (BV/TV). **(C)** Total bone surface around the implant, expressed as the index of the bone-to-implant (BIC) ratio. Sections of five groups observed and imaged via microscopy **(D)**. Hard tissue sections also reveal that the **(E)** BV/TV and **(F)** BIC of the Nell+ group were higher than the Blank group and LVNC group after 4, 8, and 12 weeks of healing. While Nell– group demonstrated the converse results. Scale bar:300 μm. *P < 0.05.

Micro-CT analysis and hard tissue section were used to evaluate the condition of implant osseointegration (Figure 6). After implantation for 4 weeks, new bone formation has been observed around the implants and within the threads. Compared to the SLA group, larger volume of newly formed bone was generated near the implants of the BMSCs sheets group. Moreover, more new bones were found around the implants of Nell+ BMSCs sheets group, and less volume of newly formed bone was generated around the implants of Nell− BMSCs sheets group (Figures 6B,E). Better-organized supporting bone around the implants of Nell+ BMSCs sheets group was formed, in contrast with the implants of Nell− BMSCs sheets group in which less bone in the peri-implant area after implantation for 8 weeks was found. The difference of newly bone formation between each group declined after implantation for 12 weeks. Nevertheless, wider contact area between bone and implants was observed in Nell+ BMSCs sheets group, while narrower bone-implant contact area was found in Nell− BMSCs sheets group. In the five groups, the Nell+ BMSCs sheets group had higher BIC and BV/TV values. However, the SLA groups had the lowest BIC values compared with other groups (Figures 6C,F).

## Discussion

This study successfully demonstrated that activation of Nell-1 significantly improves osseointegration by regulating Runx2/Osterix axis. BMSC sheets obtained from light-induce method are feasible as carriers, which are more efficient than traditional ones.

The research on osteogenic potential of Nell-1 has been carried out in recent years. It is reported that recombinant Nell-1 protein induces osteogenic differentiation in multiple MSC-like populations (Pang et al., 2015) such as murine C3H10T1/2 MSC cell line, mouse primary MSCs, and perivascular stem cells. On the contrary, Fahmy-Garcia found that Nell-1 improved vasculogenesis and MSC migration except osteoblastic differentiation (Fahmy-Garcia et al., 2018). As Nell-1 is a secreted protein, different receptors of cells binding to Nell-1 cause different biological behaviors. What’s more, protein may cause immune response in *in vivo* experiment. Therefore, our study uses lentivirus to overexpress and interfere Nell-1 gene, which obtain long-term transfection. And we deeply and directly explore the effect of Nell-1 on osteogenesis in forward and reverse. Overall, our data further supports the notion that the expression level of Nell-1 gene is positively correlated with BMSCs osteoblastic differentiation. These results are in accordance with previous studies that overexpression of Nell-1 gene promotes osteoblastic differentiation of human induced pluripotent stem cell-derived mesenchymal stem cells (Liu et al., 2014) (IPSC-MSCs) and rat BMSCs (Hu et al., 2009).

Previous *in vivo* studies mainly focused on the osteogenic potential of Nell-1 protein. It has been reported that local injection of Nell-1 protein enhances bone regeneration in femoral distraction osteogenesis model (Xue et al., 2011), calvarial defect model (Aghaloo et al., 2006), and osteoporotic animal models (Zhang et al., 2014). Aghaloo injected BMSCs transfected with adenoviral encoded Nell-1 gene into nude mice intramuscularly, and new bone formation in the treatment group, while the control group demonstrated mainly fibrotic tissue (Aghaloo et al., 2007). Our study is the first to import Nell-1 gene to implant surface to explore the effect of Nell-1 on implant osseointegration. The data suggests that Nell-1 promotes osteogenesis around implant and increases bone-implant contact area percentage. It demonstrates that Nell-1 promotes BMSCs osteoblastic differentiation and has a positive effect on implant osseointegration.

**FIGURE 7:**
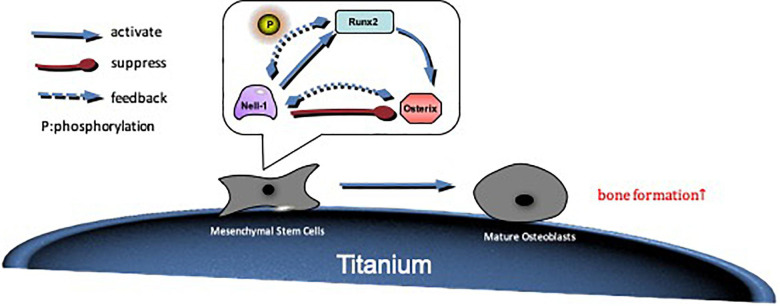
The possible mechanism by which Nell-1 regulates Runx2/Osterix axis. Nell-1 actives Runx2 and Osterix through positive feedback. The phosphorylation of Runx2 is improved, and then osteogenic differentiation of BMSCs is promoted. With the extension of time, Runx2 further improve the expression of Osterix, so that differentiation and maturation of preosteoblast are promoted.

The effect of Nell-1 gene on osteogenesis has been verified through *in vivo* and *in vitro* study, but the mechanism of Nell-1’s function on BMSCs remains unclear. Studies suggested that Nell-1 protein acted on various signaling pathway including Wnt signaling pathway, hedgehog signaling pathway, and BMP2 signaling pathway (Shen et al., 2016; Pakvasa et al., 2017; Li et al., 2018). Zhang found that Nell-1 protein induced phosphorylation of Runx2 (Zhang et al., 2011). Further studies showed that Nell-1 was a downstream regulator of Runx2 in osteogenic and chondrogenic differentiation (Li et al., 2017). Osterix is an important function factor in the process of osteogenesis, and it is one of the downstream genes of Runx2. A study showed that there were binding sites between Nell-1 and Osterix, and Osterix acted as a negative regulator of Nell-1 in Saos-2, U2OS, HeLa and Glioma cells (Chen et al., 2011). However, whether Nell-1 having an effect on the expression of Osterix or not has not been studied yet. In this study, we took Runx2 and Osterix as a signal axis, and the relationship between Nell-1 and Runx2/Osterix axis was evaluated. The results suggested that Nell-1 improved the expressions of Runx2 and Osterix at both gene and protein levels. The interference of Runx2 expression at gene level was partially rescued, but there was no significant difference. The expression of Runx2 at protein level was promoted remarkably. The reason may be that Nell-1 phosphorylates Runx2 to enhance the expression, and the expression of Runx2 is greatly reduced after the interference. Therefore, the feedback regulation is weakened. Furthermore, overexpression of Nell-1 rescued the interference of Osterix expression at gene and protein levels. In general, Runx2 up-regulates expression of Nell-1 and Osterix as an up-stream gene, and the expression of Nell-1 will be inhibited by Osterix, and Nell-1 has positive feedback regulation on the expressions of Runx2 and Osterix (Figure 7).

BMSCs are not only have the potential for osteoblastic differentiation but also can modulate RANKL-mediated osteoclastogenesis. RANKL is a multifunctional cytokine which activates NF-κB signaling pathway and promotes osteoclast formation by interacting with RANK. OPG is a decoy receptor for RANKL which prevents RANKL interaction with RANK, eventually leading to inhibition of osteoclast formation (Boyce et al., 2015). Our study suggests that Nell-1 may have negative effect on differentiation of osteoclast by regulating the OPG/RANKL expression ratio of BMSCs. However, the effect and mechanism of Nell-1 regulating the function of osteoclast is not concerned in this study, and we will focus on it in the future.

Our study provides a clear explanation of using BMSC sheets as a carrier to study the mechanism of osteogenesis. Cell sheets have been widely used to repair defects with the development of tissue engineering (Joseph et al., 2006). Compared to scaffold and traditional cells composition, cell sheets feature uniform distribution of cells and complete ECM 3-dimensional structure (Zhou et al., 2017). BMSCs are dominant cells in the process of osseointegration and have advantages of accessibility, multiple differentiation potential, and immune-evasion. Hence, BMSCs were chosen to prepare cell sheets in this study. Our data suggested that UV365 is safe for BMSC sheets harvesting. The light-controlled BMSC sheets we obtained maintained good cell viability and reattached to wells well. The results of *in vivo* study demonstrated that better osseointegration was observed in the BMSC sheets group compared to the SLA group. Interestingly, our data revealed that the bone-implant contraction of the Nell− BMSC sheets group was better than that in SLA group. The reason may be that BMSC sheets act as scaffolds which provide a structural microenvironment for cell growth, adhesion, and cell-cell conjunctions (Duan et al., 2017). Furthermore, BMSC generates more ECM around the implants. ECM can regulate cell homeostasis, differentiation, and bone regeneration around the implants (Cerqueira et al., 2014).

Fibronectin (FN) was used to promote BMSCs adhesion to quartz substrates and improve the success rate of cell sheets preparation in this study. FN is one of the components of the ECM. It was reported that FN could improve cell adhesion ability by up-regulating integrin α5β1 (Teruo et al., 2015). Integrin α5β1 is one of the transmembrane receptors between cells and the extracellular environment. It promotes the adhesion and migration of BMSCs when its expression is increased, and reduces the apoptosis of cells due to the loss of adhesion with ECM (Tao et al., 2018). In consequence, BMSCs attachment to quartz substrates with TiO_2_ nanodot films was promoted by using FN.

In this study, we successfully up-regulated the expression of Nell-1 in BMSCs, modified the implant surface through using BMSC sheets and evaluated the osteogenesis. Our study demonstrated that activation of Nell-1 enhanced osteoblastic ability of BMSCs and improved implant osseointegration via regulating Runx2/Osterix axis.

## Conclusion

The results demonstrate that activation of Nell-1 gene promotes osteogenesis through regulating Runx2/Osterix axis and enhances implant osseointegration. It is accessible to import gene to implant surface by using BMSC sheets. The research will focus on investigating the underlying mechanisms and effects of Nell-1 in regulating osteoclast activity in the future.

## Data Availability Statement

All datasets presented in this study are included in the article/supplementary material.

## Ethics Statement

The animal study was reviewed and approved by the Institutional Animal Care and Use Committee of Zhejiang University.

## Author Contributions

GY and ZJ contributed to the conception of the study. KL, YX, XD, and YL performed the experiments. KL, XM, and TH performed the data analyses and wrote the manuscript. HW and YW helped perform the analysis with constructive discussions. All authors contributed to the article and approved the submitted version.

## Conflict of Interest

The authors declare that the research was conducted in the absence of any commercial or financial relationships that could be construed as a potential conflict of interest.
